# Mobile App for Patients With Chronic Obstructive Pulmonary Diseases During Home-Based Exercise Care: Usability Study

**DOI:** 10.2196/60049

**Published:** 2024-11-15

**Authors:** Shih-Ying Chien

**Affiliations:** 1 Department of Industrial Design Chang Gung University Taoyuan Taiwan; 2 Physical Medicine & Rehabilitation Chang Gung Memorial Hospital Taoyuan Taiwan; 3 Department of Public Health & Medical Humanities School of Medicine National Yang Ming Chiao Tung University Taipei Taiwan

**Keywords:** digital health, chronic obstructive pulmonary disease, COPD, usability, telerehabilitation, mobile health app

## Abstract

**Background:**

Digital health tools have demonstrated promise in the treatment and self-management of chronic diseases while also serving as an important means for reducing the workload of health care professionals (HCPs) and enhancing the quality of care. However, these tools often merely undergo large-scale testing or enter the market without undergoing rigorous user experience analysis in the early stages of their development, leading to frequent instances of low use or failure.

**Objective:**

This study aims to assess the usability of and satisfaction with a mobile app designed for the clinical monitoring of patients with chronic obstructive pulmonary disease undergoing pulmonary rehabilitation at home.

**Methods:**

This study used a mixed methods approach involving two key stakeholders—patients with chronic obstructive pulmonary disease and HCPs—across three phases: (1) mobile app mock-up design, (2) usability testing, and (3) satisfaction evaluation. Using convenience sampling, participants were grouped as HCPs (n=12) and patients (n=18). Each received a tablet with mock-ups for usability testing through interviews, with audio recordings transcribed and analyzed anonymously in NVivo12.0, focusing on mock-up features and usability insights. Task difficulty was rated from 1 (very easy) to 5 (very difficult), with noncompletion deemed a critical error. Usability satisfaction was measured on a 5-point Likert scale from 1 (strongly disagree) to 5 (strongly agree).

**Results:**

The research indicated a notable difference in app usability perceptions: 66% (8/12) of HCPs found tasks “very easy,” compared to only 22% (4/18) of patients. Despite this, no participant made critical errors or withdrew, and satisfaction was high. HCPs completed tasks in about 20 minutes, while patients took 30. Older adults faced challenges with touch screens and scroll menus, suggesting the need for intuitive design aids like auditory support and visual health progress indicators, such as graphs. HCPs noted potential data delays affecting service, while non–native-speaking caregivers faced interpretation challenges. A secure pairing system for privacy in teleconsultations proved difficult for older users; a simpler icon-based system is recommended. This study highlights the need to consider stakeholder abilities in medical app design to enhance function implementation.

**Conclusions:**

Most HCPs (11/12, 91%) found the app intuitive, though they recommended adding icons to show patient progress to support clinical decisions. In contrast, 62% (11/18) of patients struggled with tablet navigation, especially with connectivity features. To ensure equitable access, the design should accommodate older users with diverse abilities. Despite challenges, both groups reported high satisfaction, with patients expressing a willingness to learn and recommending the app. These positive usability evaluations suggest that, with design improvements, such apps could see increased use in home-based care.

## Introduction

### Background

Chronic pulmonary diseases severely impair the respiratory system and impact the daily activities of those affected [1-4]. With approximately 3 million deaths annually, chronic pulmonary diseases rank among the top 3 global causes of death [5,6]. Chronic obstructive pulmonary disease (COPD) is a major subset of chronic pulmonary diseases, with nearly 36% of patients developing comorbidities, such as hypertension and cardiovascular diseases [7]. Globally, 1 person succumbs to COPD every 10 seconds; in Taiwan, over 5000 deaths are attributed to COPD annually [8]. Severe cases of COPD may lead to systemic manifestations, emphasizing the urgency of interventions [9,10]. Patients, particularly older adults, often struggle with dyspnea and thus experience a diminished quality of life [11]. Depressive symptoms also commonly arise along with the aforementioned problems [12,13]. The significant impact of chronic pulmonary diseases on respiratory function and overall well-being highlights the urgency of addressing this issue. COPD presents with 3 major symptoms: cough, sputum production, and wheezing and is therefore, often mistaken as the common cold [14,15]. Coupled with low awareness of obstructive lung diseases among the public, individuals frequently underestimate their condition, leading to a delayed diagnosis and treatment as well as a significantly elevated risk of health deterioration [16,17].

Exercise is recognized as one of the most effective therapies for chronic pulmonary diseases [18]. It serves to alleviate respiratory symptoms, enhance cardiorespiratory function, improve quality of life, and consequently increase the overall well-being of patients [19,20]. Statistics indicate that individuals with COPD face a reduced life expectancy of 6 to 10 years [21]. In Taiwan, the 1-year mortality rate after the first hospitalization for obstructive lung diseases is as high as 20% [22]. To prevent recurrent hospitalizations due to acute deterioration in pulmonary function, postdischarge priorities for patients with chronic pulmonary diseases include maintaining regular exercise habits and receiving precise exercise prescriptions tailored to their conditions [23,24]. This approach aims to train and enhance pulmonary capacity, thereby extending life expectancy [20,25]. To ensure that home-based pulmonary rehabilitation exercises meet clinical requirements, digital health (eHealth) tools are considered potential aids in the treatment of chronic diseases because they assist patients in self-managing their conditions [26,27]. Simultaneously, these tools can provide real-time assistance to patients, caregivers, and health care professionals (HCPs) in achieving the vision of individual disease management and monitoring [28,29]. Moreover, eHealth tools serve as the optimal instruments for HCPs to provide real-time guidance to patients at home in developing health care skills and managing chronic diseases, especially during circumstances when in-person treatments are challenging (such as during the COVID-19 pandemic) [30]. Research confirms that the application of eHealth tools not only effectively reduces incidence rates, disease exacerbation, and recurrent hospitalizations but has also proven to be an efficient means of alleviating the clinical workload of HCPs [31,32].

Chronic diseases are a primary driver of the global increase in health care and caregiving expenditures [32,33]. In this context, eHealth tools could benefit various stakeholders (including HCPs, patients, and caregivers) by offering a health service [34,35]. However, these tools often face challenges in achieving seamless operations among the stakeholders involved, leading to outcomes below expectations [36]. Consequently, patients after their discharge exhibit low adherence to using these tools, hindering the comprehensive realization of their intended benefits [37,38]. Therefore, the design of eHealth tools should prioritize a methodology centered on user experience [32,39,40].

### Objectives

For the aforementioned reasons, this study conducted a user experience–based evaluation of the usability of a home-based pulmonary rehabilitation app. The assessment focused on operational proficiency, information comprehension, interface design, and system acceptance among discharged patients and HCPs. User feedback and results were collected, and based on the survey findings, posttrial design modifications were implemented to enhance the effectiveness and user adherence to the eHealth tool. The objective was to ascertain the usability of and satisfaction with the mobile app in tracking home-based pulmonary rehabilitation exercise therapy for users.

## Methods

### Overview

This study was a nonpharmacological clinical trial that used a questionnaire-based interview approach to evaluate the operational usability of a mobile app designed for remote health care delivery. The evaluation involved 2 key stakeholder groups: the HCPs and the patients. The insights gained from this assessment will be used as a reference for refining the design of eHealth tools, ensuring their applicability for both clinical and home-based remote health care delivery.

### Ethical Considerations

This study received approval from the Institutional Review Board of the Chang Gung Medical Foundation (registration number: 202200070B0) prior to its implementation. Before the commencement of the experiment, informed consent was obtained from all participants, who signed the necessary consent forms. To protect participants' privacy, all personal information was de-identified and stored with encoded data for analysis. Throughout the data collection and processing, strict adherence to confidentiality and privacy protection principles was maintained. Furthermore, all participants voluntarily joined the study, and no monetary compensation or gifts were offered to ensure the objectivity and impartiality of the experiment.

### App Development and Usability Testing

Before initiating this study, the researchers conducted a survey on the construction requirements of a home-based pulmonary rehabilitation system. The aim was to establish the fundamental elements of system design based on user experience and needs. Building upon the outcomes of the preliminary research, 2 apps were developed: one for HCPs to clinically monitor exercise activities and another for patients or caregivers to execute the prescribed exercises at home. This study concentrated on assessing the usability of these apps for the 2 participant groups. The survey results of this study will contribute to refining the design of the home-based pulmonary rehabilitation app. The research investigation comprised the following two stages: (1) observing and recording user interactions to understand the ease of use regarding system functionalities and (2) conducting usability tests through app operations to elucidate user satisfaction with the app design. This study was conducted at the Chang Gung Hospital in Taiwan with convenient sampling. The HCP group consisted of respiratory care and pulmonary care professionals, including physicians, therapists, and nurses (n=12), who (1) were aged ≥25 years with at least 1 year of experience in respiratory or pulmonary disease care, (2) had provided care for patients with chronic pulmonary diseases in the past 3 years, and (3) had experience in tracking patient rehabilitation. The patient group comprised patients with chronic pulmonary diseases (n=18) who (1) were aged ≥58 years, (2) had been diagnosed with chronic pulmonary diseases for ≥3 years with a history of hospitalization and had experienced pulmonary rehabilitation, (3) willingly participated and signed an informed consent form, and (4) possessed the ability to express themselves independently.

**Figure 1:**
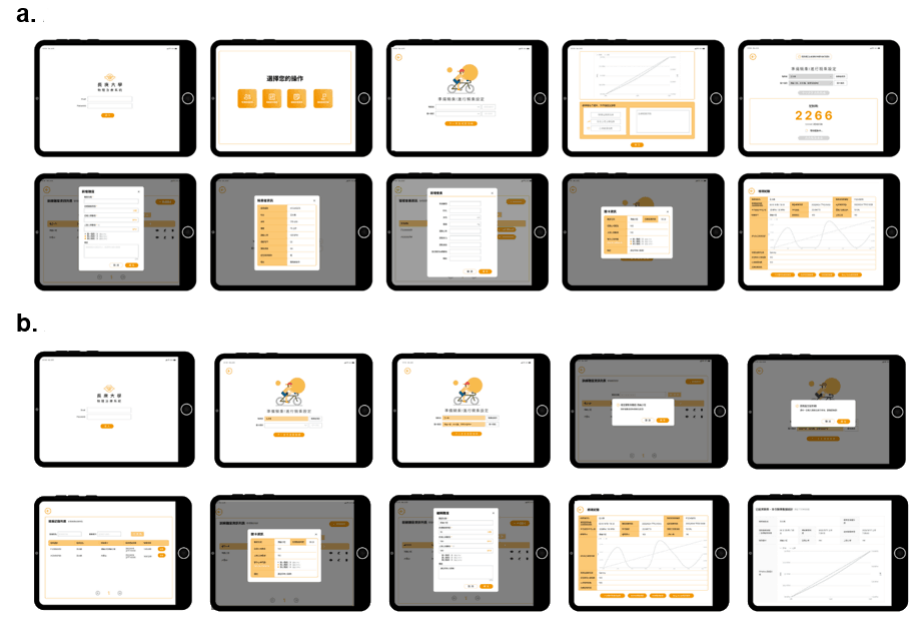
Usability test mock-ups. (A) App’s functional interface for health care professionals. Professionals can prescribe exercise regimens based on the patient’s condition, adjusting them dynamically by referencing clinical data and psychophysical states. This aids in facilitating the self-management of postdischarge patients at home. (B) App’s functional interface for patients. The interface for the home pulmonary rehabilitation app guides patients through lung exercises at home using visualizations and encompassing features, such as receiving exercise prescriptions, activity tracking, and progress monitoring.

Considering the participants were older adults, the questionnaires were administered and filled out by the trial executor. Figure 1 illustrates the main functionalities of the app and the information distribution on each screen. Usability tests were conducted on the 10 key functional pages of the app, representing the most crucial functions of the system. The details of these tasks are outlined in Textbox 1. Throughout the testing process, when participants encountered notable operational challenges, the researcher proactively inquired about their difficulties or potential consideration for discontinuation. If participants successfully accomplished a task, it was documented as “operation success” on the questionnaire. Conversely, if participants failed to complete a task in 3 consecutive attempts without adhering to instructions, the researcher categorized it as “operation failure.” Irrespective of the participants’ success, posttask feedback was collected, and a Likert scale was used to evaluate task difficulty, using the following ratings: 1=very easy, 2=easy, 3=moderately easy, 4=difficult, and 5=very difficult. To evaluate app usability satisfaction, a survey was developed by adapting and modifying validated questionnaires from previous studies to align with the objectives of this research. Regarding the satisfaction of app usability, the survey comprised 10 questions each for the HCPs and the patients (Textbox 2). Responses were assessed on a 4-point Likert scale, ranging from 1 (totally disagree) to 4 (totally agree).

**Usability test items.**

**Usability test items for assessing app functionality for health care professionals**

1. Create an account
2. Log in
3. Patient information management including entering or adding the patient’s name, age, sex, contact number, and a brief description of the patient’s physical condition
4. Prescription management: set patient prescriptions and transmit them to the patient’s end
5. Record review and analysis: view the patient’s exercise history records, including visualizing graphs showing trends in clinical signs
6. Clinical monitoring: monitor patient cycle variations, including whether the heart rate falls within the “safe heart rate” range
7. Remote monitoring: establish a connection and pair it with the patient’s end for we-based monitoring of the patient’s exercise status
8. Consultation and reporting: view exercise outcome reports and conduct web-based consultations
9. Prescription adjustment: online adjustment of exercise prescriptions, including intensity and difficulty.
10. Record management: view, edit, or delete member records

**Usability test items for assessing app functionality for patients**

1. Log in
2. Select personal information to view individual details
3. Generate a pairing code to connect with the hospital’s end
4. Retrieve exercise prescription
5. Review exercise prescription details
6. View personal exercise history records (including visualizing graphs showing trends in clinical signs)
7. Pause the exercise (can be paused at any time if discomfort is experienced)
8. Examine exercise cycle variations, including checking safe heart rate and heart rate variability
9. Receive remote connection requests for web-based consultations
10. Complete web-based questionnaires (eg, Borg Rating of Perceived Exertion scale)

**Usability satisfaction assessment of the app.**

**Response items for health care professionals**

1. Overall, this app is user-friendly.
2. The app interface is well-designed and aligns with clinical information needs.
3. On the basis of patient-generated data, the graphs are easy to interpret.
4. The app substantially assists clinical care professionals.
5. The app facilitates monitoring the home-based exercise rehabilitation of postdischarge patients.
6. Would you recommend colleagues to use a similar app?
7. The operations of the app are straightforward and easy to remember.
8. The actions performed, whether on the web or offline, are straightforward to me.
9. The app design is comprehensive with no missing or incorrect information.
10. I believe that it is safe for patients to use at home.

**Questions for patients**

1. Overall, this app is user-friendly.
2. The app has a simple and easy-to-understand interface.
3. The app facilitates easy recall of exercise prescription information at home.
4. The app comprehensively records the entire exercise process, ensuring no loss of vital information.
5. When encountering issues, I can easily resolve them.
6. Virtual reality contributes to my increased focus and enjoyment during rehabilitation.
7. I intend to continue using this app.
8. Even without assistance, I can operate the app on my own.
9. I feel safe using this app at home.
10. I find some features a bit complex.

In addition, to ensure the accuracy of the participants’ feedback, the entire experimental process was recorded with meticulous time control to ensure that each participant completed the interview within the designated time frame (50-60 minutes). Participants had the opportunity to express their perspectives, opinions, and experiences regarding the tasks during the experiment (such as the difficulties or simplicity in the task). Subsequently, this information underwent verbatim transcription analysis with relevant usability keywords marked. Detailed information on participants’ sociodemographic and clinical characteristics can be found in subsequent sections. Furthermore, the quantitative data of this study were analyzed using the statistical software SPSS (version 22.0; IBM Corp).

## Results

### Overview

This study collected data from two groups: HCPs (n=12) and patients (n=18). The majority (8/12, 66%) of HCPs were respiratory therapists with an average age of 46 (SD 5) years. They had >1 year of experience in respiratory care within the past 3 years. The majority (16/18, 89%) of patients with chronic lung disease were male, with an average age of 66 (SD 5) years. They had a history of chronic lung disease for >3 years and had undergone pulmonary rehabilitation for ≥3 years. Detailed information regarding the sociodemographic and clinical backgrounds of these two participant groups can be found in Table 1.

**Table 1:** Sociodemographic characteristics of participants.

Participants and characteristics				Values
**HCPs^a^ (n=12)**				
	Age (y), mean (SD)		46 (5)	
	**Sex, n (%)**			
		Male	3 (25)	
		Female	9 (75)	
	**Job title, n (%)**			
		Respiratory therapist	8 (67)	
		Thoracic surgeon	1 (8)	
		Physiotherapist	1 (8)	
		Pulmonary rehabilitation specialist	2 (17)	
	**Experience in caring for chronic respiratory diseases (y), n (%)**			
		1-3	3 (25)	
		>3	9 (75)	
**Patients (n=18)**				
Age (y), mean (SD)				66 (5)
	**Gender, n (%)**			
		Male	16 (89)	
		Female	2 (11)	
	**Education level, n (%)**			
		Elementary	10 (56)	
		High school	3 (17)	
		Bachelor	3 (17)	
		Bachelor’s degree or higher	2 (11)	
	**Duration of illness (y), n (%)**			
		1-3	5 (28)	
		>3	13 (72)	

**^a^**HCP: health care professional.

### User Operations and Usability Perception Interview Survey

Following user interaction with the system, we conducted one-on-one semistructured interviews to gather insights into operational experiences. The participants described any difficulties encountered as well as their feelings while executing the tasks. The entire interview process was recorded, transcribed verbatim, and then subjected to qualitative analysis using NVivo (version 12.0; Lumivero) for content analysis and synthesis.

Among the HCPs, 66% (8/12) acknowledged the need for a brief transitional period to familiarize themselves with the app interface and functionalities. During the transformation of data into graphical representations, more diverse visualizations were preferred. Specifically, 58% (7/12) expressed a preference for observing changes in heart rhythm and having graphical representations illustrating cyclic patterns to aid in explaining the progression of pulmonary function in patients.

Moreover, 84% (10/12) of the HCPs emphasized from the hospital’s standpoint, the concern regarding inadequate self-health management in patients after discharge, especially considering that these patients belonged to a high-risk demographic and reported that prioritizing safety should be a top consideration. Therefore, it is recommended that the fundamental design of the app incorporate an emergency cessation mechanism or a real-time notification feature that activates upon detecting physiological abnormalities, aligning with the fundamental requisites for medical applications.

Within the patient participant group, 83% (15/18) of respondents reported a sense of complexity during their initial exposure to the app. Among them, 62% (11/18) indicated an inability to operate the app independently; this challenge was attributed to the age of the majority of participants (mean 66, SD 5 years) and their inherent skepticism regarding their operational capabilities. Despite the user interface of the patient-side app being menu-based and devoid of text input requirements, using tablets and apps as interactive media was still perceived as challenging. Moreover, 45% (8/18) expressed difficulty in discerning or comprehending the information presented on the screen, contributing to feelings of unease and anxiety; 62% (11/18) believed that understanding the operational procedures of the app without guidance was challenging. Notably, the majority (12/18, 67%) of patients specifically highlighted the difficulties encountered during the initial step of entering email and password information to log in to the system. Lastly, 39% (7/18) conveyed an inability to comprehend the numerical representations in the postexercise feedback reports, expressing curiosity or confusion regarding the meaning of the graphs. Table 2 presents detailed feedback from participants.

**Table 2:** Operational challenges: findings from the semistructured interview.

Participant and participant’s feedback			Pain points
**HCPs^a^**			
	“Um... Honestly, at first, without any instructions, I was a bit unsure where to start, but luckily, I quickly found the “+” sign to create an account.” [HCP 6]	The “+” symbol for account creation might not be very clear, but fortunately, it was quickly resolved.	
	“I found it easy to fill in basic patient information, but prescribing medication is more challenging for me since I’m not a doctor.” [HCP 1]	Different backgrounds may entail different responsibilities.	
	“I’m not sure if it’s just me being unfamiliar with the system, but currently, while the screen displays patient heart rate and related data well, for clinical staff, besides linear charts illustrating historical backgrounds, it would be helpful to have icons indicating categories or different charts displaying changes in various physiological values for clarity.” [HCP 11]	Clinical staff require more varied graphical representations or symbols to express the significance of diverse clinical data.	
	“I’m not sure if it’s a network issue or a system problem, but I feel the screen updates a bit slowly.” [HCP 4] “Perhaps there’s some network delay; when I modify prescriptions, the patient’s screen doesn’t always match what I intend to prescribe.” [HCP 12]	It is essential to ensure data conversion speed and the ability to promptly provide accurate information.	
	“Hmm... Typically, it takes some time to accumulate clinical data to see the effectiveness. At this stage, although the feature exists, there might not be enough data to discern changes.” [HCP 1]	A vast amount of data are required for clinical efficacy to be evident in the system.	
	“I’m afraid I might accidentally delete a patient’s record.” [HCP 5]	There is a lack of mechanism for recovery or error compensation.	
**Patients**			
	“I can’t read, and I can’t see what’s written on the screen.” [Ps 8 and Ps 17]	Patients who are older adults have lower levels of education or have poor vision and may not be comfortable with operating the app alone.	
	“I can use a tablet, but I don’t know how to type.” [Ps 17]	In addition to typing, other input functions and voice commands need to be added.	
	“I don’t really understand what these numbers mean...And what is pairing connection...Do I just press it, or do I need to input something?” [Ps 5, Ps 9, and Ps 12]	The connection mechanism poses a challenge for both the older adults and caregivers.	
	“I often accidentally press the pause button... I don’t know how to get back to the exercise screen...It makes me very anxious.” [Ps 13]	There are too many function keys on the screen, making it difficult for users to navigate.	
	“I see my heartbeat and heart rate...I don’t quite understand them, so it would be better if there were colors or lines to remind me when to slow down.” [Ps 10]	The interface should be more user-friendly to avoid excessive use of numbers and scientific charts and should make good use of patterns, icons, or voice commands.	
	“I received a message asking to connect and to input numbers. I find this very difficult.” [Ps 8]	Text may be difficult to understand; replacing it with diagrams or call-in features might be easier to comprehend.	
	“This thing is too advanced; I can’t figure it out.” [Ps 7]	The digital divide is a significant barrier for many rural and older adult populations.	

^a^HCP: health care professional.

### Evaluation of User Operational Difficulty

During the assessment of the usability of the app’s function, HCPs encountered minimal challenges in tasks, such as setting up user and patient accounts as well as modifying patient information using tablets. Specifically, 8 (67%) of the 12 participants rated these tasks as “very easy.” More than half (7/12, 58%) of the HCPs found it easy to set and transmit prescriptions to the patient’s end. In addition, the majority (9/12, 75%) found it relatively straightforward to access patient exercise histories and visualize health status charts, although some recommended potential enhancements.

Concerning the web-based monitoring of changes in patient physiological readings, the majority (9/12, 75%) of HCPs perceived the interface to be clear and easy to understand, with operations being very straightforward. However, when establishing connections, most (6/12, 50%) participants initially found it somewhat difficult. Nevertheless, after becoming familiar with the process, they regarded it as relatively simple (8/12, 66%).

While tasks like adjusting web-based prescriptions and conducting remote consultations were found easy by most (9/12, 75%) HCPs, 7 (58%) of the 12 HCPs expressed that accessing patient information and engaging in web-based consultations were comparatively complex, requiring more time for comprehension.

Managing patient information was deemed straightforward by 10 (83%) of the 12 HCPs. They noted that the clear interface facilitated easy access to and deletion of information. Screen delays were reported by only 42% (5/12) of participants, classifying this issue as “moderate” (Table 3). Importantly, no task was rated as “difficult” or “too difficult.”

**Table 3:** Perceived difficulty of participant operations.

Participant and task			Very easy, n (%)		Easy, n (%)		Moderately difficult, n (%)		Difficult, n (%)		Very difficult, n (%)
**HCPs^a^ (N=12)**											
	T1: Create an account	8 (67)		3 (25)		1 (8)		0 (0)		0 (0)	
	T2: Log in	8 (67)		3 (25)		1 (8)		0 (0)		0 (0)	
	T3: Patient information management	8 (67)		4 (33)		0 (0)		0 (0)		0 (0)	
	T4: Prescription management	4 (33)		7 (58)		1 (8)		0 (0)		0 (0)	
	T5: Record review and analysis	3 (25)		9 (75)		0 (0)		0 (0)		0 (0)	
	T6: Clinical monitoring	2 (17)		9 (75)		1 (8)		0 (0)		0 (0)	
	T7: Remote monitoring	2 (17)		8 (67)		2 (17)		0 (0)		0 (0)	
	T8: Consultation and reporting	3 (25)		9 (75)		0 (0)		0 (0)		0 (0)	
	T9: Prescription adjustment	3 (25)		4 (33)		5 (42)		0 (0)		0 (0)	
	T10: Record management	2 (17)		10 (83)		0 (0)		0 (0)		0 (0)	
**Patients (N=18)**											
	T1: Log in	4 (22)		8 (44)		1 (6)		5 (28)		0 (0)	
	T2: Viewing personal information	5 (28)		6 (33)		3 (17)		4 (22)		0 (0)	
	T3: Pairing for connection	4 (22)		3 (17)		5 (28)		6 (33)		0 (0)	
	T4: Retrieving exercise prescription	6 (33)		3 (17)		4 (22)		5 (28)		0 (0)	
	T5: Reviewing prescription details	6 (33)		11 (61)		1 (6)		0 (0)		0 (0)	
	T6: Viewing past exercise history	6 (33)		11 (61)		1 (6)		0 (0)		0 (0)	
	T7: Pausing exercise	12 (67)		6 (33)		0 (0)		0 (0)		0 (0)	
	T8: Examining cycle variations	5 (28)		7 (39)		4 (22)		2 (11)		0 (0)	
	T9: Requesting web-based consultations	1 (6)		2 (11)		7 (39)		8 (44)		0 (0)	
	T10: Completing questionnaires on the web	3 (17)		15 (83)		0 (0)		0 (0)		0 (0)	

In contrast, the patient group experienced substantial challenges during system log-in. Most (6/18,34 %) patients initially struggled to understand how to use the tablet. After reminders and demonstrations, 45% (8/18) of the patients eventually considered the log-in process as “easy,” while 28% (5/18) found it challenging. When viewing personal information, 34% (6/18) individuals expressed that it was relatively simple, despite requiring some time for searching and consideration.

Regarding receiving pairing codes and connecting to the hospital end, most (7/18, 39%) patients could input the pairing code to establish a connection; however, 34% (6/18) still encountered difficulties due to a lack of familiarity and the absence of assistance from caregivers. Despite some (9/18,50 %) patients expressing confusion regarding the functionality on the screen to receive prescriptions from HCPs, 33% (6/18) and 17% (3/18) patients found it “very easy” and “easy,” respectively.

In addition, concerning viewing exercise prescriptions and accessing personal exercise history records (including visualized charts displaying clinical symptom trends), the majority (11/18, 61%) of patients considered these tasks relatively simple. When experiencing discomfort, 67% (12/18) knew which button to press to pause and considered this step as “very easy.”

Regarding viewing personal physiological information, 7 (39%) of the 18 patients found it relatively easy; however, 8 (44%) patients expressed uncertainty about how to initiate web-based consultations. Nonetheless, although participants within the patient group encountered operational difficulties, none described the tasks as “too difficult” in the questionnaire interviews (Table 3).

### Usability Testing of System Task Accomplishment

In usability testing with HCPs, system tasks, such as setup, log-in, exercise prescription, and remote connectivity were assessed. None of the tasks were completely successful on the first attempt. However, log-in and clinical monitoring tasks showed a higher success rate, reaching 83% (10/12; Tables 4 and 5). In the testing process, each participant was given 5 opportunities for operation. The majority (8/12, 66%) of HCPs required some time to adapt to and familiarize themselves with the app features. About 42% (5/12) committed 1 to 2 errors during operations, while 25% (3/12) made 3 or more errors. Nevertheless, 58% (7/12) ultimately succeeded in completing the tasks within the specified time limit. The HCPs attributed these errors primarily to the unfamiliarity with the interface and the occasional accidental presses. However, they noted that the design of the system was not overly complex, and the inclusion of a “back” mechanism allowed for quick error correction.

**Table 4:** Success rates of participant actions (N=122).

Task	Success, n (%)	Failure, n (%)
T1: Create an account	9 (75)	3 (25)
T2: Log in	10 (83)	2 (17)
T3: Patient information management	8 (66)	4 (33)
T4: Prescription management	8 (66)	4 (33)
T5: Record review and analysis	9 (75)	3 (25)
T6: Clinical monitoring	10 (83)	2 (17)
T7: Remote monitoring	8 (66)	4 (33)
T8: Consultation and reporting	7 (58)	5 (42)
T9: Prescription adjustment	8 (66)	4 (33)
T10: Record management	9 (75)	3 (25)

**Table 5:** Success rates of participant (patient) actions (n=18).

Task	Success, n (%)	Failure, n (%)
T1: Logging in	8 (44)	10 (56)
T2: Viewing personal information	9 (50)	9 (50)
T3: Pairing for connection	6 (33)	12 (67)
T4: Retrieving exercise prescription	9 (50)	9 (50)
T5: Reviewing prescription details	11 (61)	7 (39)
T6: Viewing past exercise history	13 (72)	5 (28)
T7: Pausing exercise	16 (89)	2 (11)
T8: Examining cycle variations	10 (56)	8 (44)
T9: Requesting web-based consultations	4 (22)	14 (78)
T10: Completing questionnaires on the web	18 (100)	0 (0)

Conversely, the majority (12/18, 67%) of the patients were able to complete tasks with assistance. However, tasks such as “Pairing for connection” and “Request web-based consultations” remained challenging for many, with failure percentages of 67% (12/18) and 78% (14/18), respectively. Among these patients, 44% (8/18) made 1 to 2 mistakes, while 39% (7/18) made 3 or more errors on the test (Table 5).

During the study, the main reason for errors among the patients was the fact that they were older adults, which may have affected their ability to operate the tablet as we would have expected. Notably, the patients achieved a 100% success rate in Task 10, “Completing questionnaires on the web,” primarily because this functionality was operated by the HCPs at this stage, thereby encountering fewer issues in operation.

Moreover, the experiment was conducted in a medical setting; as a result, none of the participants in either group made critical mistakes.

### Mobile App Satisfaction Survey

Regarding the satisfaction survey, over half (11/12, 91%) of the HCPs expressed satisfaction with the functionality design of the app, finding it relatively satisfactory with minimal operational issues. A substantial proportion, constituting 75% (9/12), perceived the app as highly user-friendly, and 66% (8/12) expressed considerable satisfaction with the design of the interface. Moreover, the HCPs could promptly access patients’ physiological information in real time through the app, with 58% (7/12) indicating such capability. However, satisfaction levels slightly declined when monitoring patients’ physiological conditions through web-based connectivity. Only 33% (4/12) perceived this function as comprehensive, while 17% (2/12) expressed concerns regarding the inability of the current connection quality to facilitate real-time monitoring. Furthermore, 25% (3/12) suggested that a more stable network connection would enhance safety. Finally, the extraction of information and the generation of graphical representations were considered crucial by most (11/12,91%) HCPs. In this study, 42% (5/12) and 33% (4/12) HCPs expressed extreme satisfaction and satisfaction, respectively, with the design of this app. Respondents anticipated that these functionalities would aid HCPs in assessing patients’ physical conditions and prescribing medical treatments.

In the patient group, satisfaction with operational aspects notably lagged behind that of the HCPs across functions, such as operation, information retrieval, connectivity, and message access. Only 22% (4/18) of the patients perceived the interface design of the app as user-friendly, with nearly 33% (6/18) unable to provide a proper evaluation. This is attributed to the significant operational challenges faced by the patients, with 50% (9/18) of the users unable to comprehend each interface function (neutral 5/18, 28%, disagree 2/18, 11%, and strongly disagree 2/18, 11%). Moreover, a high percentage (13/18, 72%) were unaware of how to connect remotely, and 39% (7/18; neutral 2/18, 11%, disagree 4/18, 22%, and strongly disagree 1/18, 6%) were unsure of how to access historical rehabilitation exercise records. In addition, 61% (11/18) were uncertain about accessing personal messages, and over a quarter (5/18, 28%) expressed confusion about the significance of real-time values. Nonetheless, 61% (11/18; strongly agree 6/18, 34% and agree 5/18, 28%) expressed willingness to recommend and continue using the app (Table 6).

**Table 6:** App satisfaction survey.

Participant and task		Strongly agree, n (%)	Agree, n (%)	Neutral, n (%)	Disagree, n (%)	Strongly disagree, n (%)
**HCPs^a^ (n=12)**						
	Easy to use	9 (75)	2 (17)	1 (8)	0 (0)	0 (0)
	User-friendly interface	8 (67)	3 (25)	1 (8)	0 (0)	0 (0)
	Well-designed patient information management	6 (50)	3 (25)	1 (8)	2 (17)	0 (0)
	Effective real-time patient data access and analysis	7 (58)	4 (33)	1 (8)	0 (0)	0 (0)
	Effective web-based health monitoring functionality	4 (33)	4 (33)	2 (17)	2 (17)	0 (0)
	Clear medical information and graphics	5 (42)	4 (33)	1 (8)	2 (17)	0 (0)
	Well-implemented connectivity features	4 (33)	4 (33)	1 (8)	3 (25)	0 (0)
	Data aiding decision on rehabilitation prescription	3 (25)	4 (33)	3 (25)	2 (17)	0 (0)
	Easy web-based prescription setup and adjustment	4 (33)	5 (42)	1 (8)	2 (17)	0 (0)
	Convenient record retrieval	4 (33)	5 (42)	1 (8)	2 (17)	0 (0)
**Patients (n=18)**						
	User-friendly interface	4 (22)	3 (17)	6 (33)	4 (22)	1 (6)
	Easy access to personal information	5 (28)	3 (17)	4 (22)	4 (22)	2 (11)
	Understanding of each feature on the interface	4 (22)	5 (28)	5 (28)	2 (11)	2 (11)
	Easy-to-use connectivity features	2 (11)	3 (17)	5 (28)	4 (22)	4 (22)
	Convenient access to historical records	7 (39)	4 (22)	2 (11)	4 (22)	1 (6)
	Well-designed personal prescription collection and execution	5 (28)	4 (22)	4 (22)	4 (22)	1 (6)
	Implementation of security mechanisms	6 (33)	5 (28)	2 (11)	4 (22)	1 (6)
	Personal exercise variations incorporated	5 (28)	3 (17)	5 (28)	3 (17)	2 (11)
	Complete personal message records	3 (17)	4 (22)	6 (33)	4 (22)	1 (6)
	Willingness to recommend this app	6 (33)	5 (28)	4 (22)	3 (17)	0 (0)

^a^HCP: health care professional.

## Discussion

### Principal Findings

This study has revealed a dichotomy in app operation and satisfaction levels between the participating HCPs and patients. Both groups exhibited a demand for adapting to the app, with the majority (11/12, 91%) of the HCPs potentially acquiring app operation skills and understanding its functions through learning and adaptation. Conversely, some (9/18, 50%) participants in the patient group found it challenging to use tablets to assist them in rehabilitation programs. Overall, there exists a correlation between operational capability and satisfaction levels. Although both groups of participants provided suggestions for the app, they all recognized that the use of this app would contribute to personal health management and remote home health monitoring. In addition, the results of the usability tests and the suggestions provided will aid us in devising improvements to the design of the mobile app.

### Considering Human Factors for Enhancing Health Care App Usability Design

Technological advancements offer various methods to enhance health care service quality. Despite the longstanding application of digital technology in medical facilities and home-based care, the practical implementation and user acceptance of eHealth tools remain limited [41]. A key reason is the absence of user-centric interface design [42]. A 2024 study highlights the importance of considering all stakeholders’ perspectives and needs in product, system, and service design, identifying the significant challenge posed by the absence of such a focus on digital health care technology [32]. The study reveals that software usability directly impacts the smooth delivery of health care products and services and determines users’ operational capability, acceptance, and satisfaction [43].

Our research indicated that the principle of user-centric design can be further refined to tailor designs to different stakeholders. Regarding the app interface proposed in this study, some HCPs required a short period to learn and adapt to the app to assess whether its functionalities met their needs or required improvement. Similarly, many patients appeared to lack the skills and abilities to sufficiently manage eHealth technology; therefore, they were possibly unable to provide effective evaluations on functionality evaluations or suggestions for improvement or even determine the potential of the app for future home use. Previous research has emphasized that the challenges arising from implementing home-based care often stem from overly complex, expensive, or bulky equipment [44]. This study builds upon current literature and suggests that the technology used at home could pose challenges for patients who are older adults [44]. One challenge is attributed to issues with interface design between software and hardware, which negatively impact performance [45]. Thus, home-based care devices and equipment could be perceived as unhelpful and introducing such technology into patient rehabilitation at home could be rendered futile. Consequently, interface design should prioritize not only the capabilities and preferred modes of communication of app users but also how the information is transmitted and visually displayed. For instance, considering the educational levels of older adults and their unfamiliarity with consumer electronics, graphical representations should be favored over text and clickable options preferred over dropdown menus [46].

### Feasibility of Digital Technology in Home Health Care

The benefits of digital technology alleviate the workload of clinical personnel in addition to providing more precise references for clinical decision-making [47]. An immediate assessment of patients’ current physiological status and a prediction of their needs can be derived from the data generated by user interaction behavior [48]. However, effective information communication and exchange rely on the capabilities of caregiver and patients. Therefore, a well-designed user interface becomes a crucial element in message transmission and is also a reason for users to accept, trust, and rely on technology [49].

Or et al [50] explored technology acceptance among patients with early-stage disease, with a primary focus on factors relevant to older adult populations. The authors suggested that patients are more likely to adopt technologies if they perceive them as useful or are satisfied with the recommendations provided by HCPs through technological assistance. Conversely, users may reject or discontinue communication with HCPs when they fail to recognize the benefits of technology, struggle to understand its significance due to technological barriers, or experience negative emotions, such as increased anxiety, uncertainty, or fear.

The feasibility and acceptance of technology for managing chronic diseases among older adults entail key considerations, primarily due to potential limitations in perceptual abilities [51]. Factors such as diminished cognitive function, tactile sensitivity, and visual acuity may hinder the use of technology, consequently impacting the efficacy and acceptance of using mobile apps. Research findings indicate significant challenges among most older individuals in navigating touch-screen controls alongside difficulties in comprehending textual information displayed on screens. Hence, presenting relevant information through visual representations or incorporating voice prompts better caters to the needs of the older adults.

On the contrary, both patients and HCPs with higher education levels and tablet familiarity preferred using visual aids instead of text to illustrate patient recovery progress. In addition, they advocated for longer data cycles and considered them instrumental in enhancing the accuracy of medical decisions and influencing patient recovery results and timelines. As irreversible conditions, chronic lung diseases necessitate long-term monitoring and health management, making home rehabilitation and health management an inevitable trend among patients. The use of digital technology not only benefits HCPs but also closely relates to the health of patients with chronic disease. However, the benefits of digital technology can only be realized by leveraging its functional advantages, which allows all parties involved to benefit from and consequently accept eHealth tools. The usability tests conducted in this study revealed that despite numerous suggestions and challenges encountered by both groups of participants in using the app, there was still a high level of satisfaction with the intervention method. Importantly, most (11/18, 61%) participants expressed willingness to adopt this approach as a future method of health management.

### Recommendations for App Design Based on the Results of Usability Testing

The usability study revealed challenges for older adults in using tablets or other touch-screen interfaces [52]. In addition to lower-than-expected tactile responses, their limited visual acuity and cognitive abilities may hinder their understanding of on-screen information [53]. Some studies have suggested that presenting information in visual formats is an effective method to alleviate communication barriers and enhance comprehension, particularly benefiting older adults [54]. Those with limited tactile perception may require additional aids such as auditory cues or styluses.

Both participant groups in this study recommended that designs be easily understandable and advocated for presenting clinically relevant graphical data in a simple and intuitive manner. Furthermore, during the trial period, some patients struggled with input commands (eg, drop-down menus) due to unfamiliarity with tablet touch-screen interfaces, leading to feelings of helplessness or confusion. Consequently, such input interfaces may not be suitable for older adults, and alternatives are required to meet their needs, for example, click-based interactions coupled with imagery, which are widely used pain assessment tools.

In terms of operational capability, most (11/12, 91%) HCPs encountered a few issues in tasks such as creating account, logging in, accessing patient information, managing or adjusting web-based prescriptions, and monitoring patient dynamics. However, some HCPs expressed concerns about the impact of connection quality on delivering medical services. Unstable connections could lead to message asynchrony as well as screen delays and lagging. While some HCPs considered this a normal occurrence due to data processing and screen updates, others believed that it might affect the effectiveness of medical services.

In contrast, patients could face more challenges in operation. Research observations have indicated that the primary reason that most patients can operate technology smoothly is due to assistance from caregivers. However, as the interface of this app is primarily displayed in Chinese, caregivers whose native language is not Chinese likely require additional explanations and perhaps training to understand the app. To address this issue, future improvements could consider using icons to replace textual instructions for broader user adaptation.

To enhance patient privacy and security, the app implemented a bidirectional web-based consultation feature. However, this feature may not be user-friendly for patients, as many of them are unfamiliar with how to input pairing connection values via touch screens on tablets. Consequently, even if the hospital sends a request message, patients may struggle to comprehend or operate this feature. To address this issue, it is suggested that a simpler approach be adopted, such as using a basic incoming call icon as a prompt for physician calls or as a signal for connection.

Our findings demonstrate that placing human-centered design at the forefront is paramount. Considering the abilities, cognition, and perceptions of different stakeholders will aid in improving the design, usability, and acceptance of eHealth apps. Throughout the study period, no critical mistakes, such as incomplete tasks, were reported by the patients and the HCPs. Moreover, a high degree of satisfaction was expressed. Despite significant operational challenges faced by the patients, they indicated a willingness to learn to use the app and recommend it to others. This suggests that such a model has the potential to be used in home-based care settings; however, further considerations in design details are necessary.

This study involved 2 participant groups and aimed to assess the usability, cognition, and acceptance of eHealth interfaces from their perspectives. The study included a total of 30 participants, comprising 12 (40%) HCPs and 18 (60%) patients; this met the required sample size for such studies. Nielsen [55] suggested that 5 participants are typically adequate to identify most issues; however, the actual sample size may vary depending on different categories, which has also been noted by numerous related studies. The findings of this study are consistent with those of other investigations evaluating the usability of eHealth technology tools. Nonetheless, there is a need for further refinement, particularly in simplifying the communication hardware and app interfaces used by patients. Tablets may not be the most suitable tools for older adults, and other alternative solutions such as voice calls instead of text input should be considered.

### Conclusions

The survey results of this study indicated that most (11/12, 91%) HCPs found the app intuitive and easy to use, while most (5/18, 28%) older patients found it challenging to operate independently. Nevertheless, both groups of participants exhibited a high level of satisfaction in the usability satisfaction survey. It is imperative for us to focus more on discussing and addressing the difficulties and dissatisfactions encountered by participants during app use to enhance the potential for future home-based apps. By examining errors during actual operations, conducting usability satisfaction surveys, and analyzing qualitative data, we can gain a better understanding of the use of mobile health apps during testing. The results from user testing aided in comprehending the actual functioning of mobile apps and served as a basis for design modifications. Moreover, our usability tests underscore the importance of tailoring app designs to accommodate contextual factors and user characteristics, such as age, education, and functional conditions, especially in populations with chronic diseases like COPD. Addressing these variables is pivotal for ensuring the effective adoption and acceptance of digital technologies in health care settings.

## Abbreviations

COPD: chronic obstructive pulmonary disease
HCP: health care professional

## Data Availability

The datasets used in this study are not publicly accessible due to the need to safeguard participants’ personal data. However, interested parties may obtain access to the datasets by contacting the corresponding author and making a reasonable request.
